# Independent secondary dose calculation for patient‐specific quality assurance: Quantitative benefit of Monte–Carlo and custom beam modeling

**DOI:** 10.1002/acm2.70265

**Published:** 2025-09-23

**Authors:** Lone Hoffmann, Mai‐Britt Linaa, Ditte Sloth Møller

**Affiliations:** ^1^ Department of Oncology Aarhus University Hospital Aarhus Denmark; ^2^ Department of Clinical Medicine Faculty of Health Aarhus University Aarhus Denmark

**Keywords:** action level, dose computation, independent secondary dose computation, Monte Carlo, patient specific quality assurance

## Abstract

**Background:**

Independent secondary dose calculation (ISDC) is becoming increasingly important for patient specific quality assurance. The most widely used analytical algorithms in ISDC are becoming challenged by Monte Carlo systems, which offer a potentially higher accuracy.

**Purpose:**

Quantify the benefit of Monte Carlo over analytical algorithms, and of customized beam models over generic beam models, in terms of clinically relevant parameters, action level, and workload.

**Methods:**

A set of 100 patients across 20 case classes, all planned with Acuros XB (Siemens Healthineers) was analyzed with Mobius3D (M3D) (Siemens Healthineers) and SciMoCa (Radialogica LLC), both with custom beam models (SMCcbm) and generic beam models (SMCgen). Gamma pass rate (GPR) and mean target dose difference |ΔD| action levels were determined for various rates of QA failures.

**Results:**

At a workload of < 10%, the action level for M3D was GPR (3%, 3 mm) < 90% and |ΔD| > 4.5%. For SMCgen, the action level was GPR (2%, 2 mm) < 95% and |ΔD| > 1.5%. For SMCcbm, it was GPR (2%, 1 mm) < 95% and |ΔD | > 1%. The combination of both criteria reduced the workload to < 5%. SMC failures could be traced back to differences in the patient density model of Acuros XB. Some M3D failures could be traced back to the handling of tissue heterogeneities. The different performance between SMCcbm and SMCgen was due to one (of three) generic beam models.

**Conclusion:**

Monte Carlo allows substantially stricter acceptance criteria and is sensitive enough to capture TPS commissioning errors. Generic beam models must be validated thoroughly before being put to use in ISDC.

## 1 Introduction

The constant evolution and diversification of radiotherapy treatments has led to an ever‐growing complexity of workflows and treatment planning tools, requiring effective patient‐specific quality assurance (PSQA). One such QA method of the treatment planning process, independent secondary dose calculation (ISDC), which incorporating accurate dose computation algorithms, has been recommended by the ICRU 83 and the AAPM TG 219 reports.^1^, ^2^ The premise of effective ISDC is two‐fold: (I) the accuracy of the employed algorithms needs to match or exceed that of the treatment planning software;^1^ (II) the treatment plan needs to be validated via clinically relevant criteria defined for the patient anatomy.

At first glance, a more accurate and meticulously commissioned ISDC software raises the sensitivity to detect errors, such as overly modulated treatment plans, problematic TPS commissioning or faults in the construction of the patient density model. The sensitivity of a QA method is also a consequence of the action level, which defines what can be seen as a tolerable slip and what constitutes a significant incident. In practice, a picky action level results in a gratuitously high workload by producing false positives, whereas a more lenient one could risk serious mistreatment. More often than not, the rate of false positives—and thereby the workload —dictates—a workable action level.

False positives are caused by the inaccuracy of the software solutions employed in both the treatment planning system (TPS) and ISDC. If the planned and recalculated dose distribution disagree, it could be a foible of the TPS, one of the ISDC, or both. Hence, the most effective means to cut the rate of false positives is high overall accuracy of the ISDC, supposing that the TPS operates at its optimum. If it does not, a highly accurate and well commissioned ISDC may reveal the TPS as the cause of frequent false positives at a sensitive action level.

The question we explore here is how the inaccuracy of an ISDC bears on a workable action level. In theory, the action level can be derived from measurements of sensitivity and specificity, which requires the knowledge of the true fails in the samples.^3^ In radiotherapy practice, such ground truth is usually inaccessible (or established by decree). Hence, we suggest the following method: for a representative collection of clinical cases, first establish the action level that results in a certain plan failure rate. Then, compare various ISDC methods via the action level they permit for a given failure rate. In other words, we search for the most sensitive action level, that is, still compatible with clinical circumstances.

The ISDC solutions compared here are deliberately chosen to represent different concepts. The Mobius3D (Varian Medical Systems, Palo Alto, USA) software incorporates a convolution–superposition dose computation algorithm with generic beam models for the Varian TrueBeam linear accelerators employed in this study (M3D).^4^ The SciMoCa (Radialogica LLC, St. Louis, USA) software comprises a Monte Carlo algorithm paired with either generic (SMCgen) or custom‐commissioned beam models (SMCcbm).^5^, ^6^ Both products support gamma evaluation and various dose‐volume metrics for plan analysis. This allows us to quantify the difference between a type B algorithm (M3D) and a type C^1^ algorithm (SMC, Acuros) as well as the difference between generic (SMCgen) and custom beam models (SMCcbm).^7^, ^8^, ^9^

## 2 Methods and Materials

### 2.1 Patient data, treatment planning, and TPS dose calculation

A total of 100 patients were drawn consecutively from the clinical database, comprised of 20 case classes with five patients per case class (H&N, lung, lung SBRT/IMRT, lung SBRT/static‐conformal, esophagus, breast, breast+SIB, breast supraclav+DIBH, spine met, brain SRT, rectum, cervix, cervix+pelvicLN, prostate+pelvicLN, brain, sinonasal, sarcoma arm, sarcoma leg, prosthesis, and palliative). These cases represent a broad range of patient groups treated in our department, with different treatment techniques and levels of challenges posed by treatment volumes and locations. For all patients except the lung and breast supraclav+DIBH cases, a free‐breathing CT scan was acquired. For lung cancer patients, the mid‐ventilation phase of a four‐dimensional (4D) CT scan was used for delineation and dose calculation. For breast supraclav+DIBH, a CT scan performed in deep inspiration breath hold was utilized. A slice thickness of 3 mm was used for all cases except for H&N (2 mm), spine mets (1.5 mm), and brain SRT (1.5 mm).

The patients were treated using Varian TrueBeam linacs (Varian Medical Systems, Palo Alto, CA, USA) equipped with a Millenium or MilleniumHD 120 multileaf collimator (MLC). The beam qualities were 6 MV, 6 MV FFF, and 15 MV. All treatment plans were created in the Eclipse TPS (Varian Medical Systems, Palo Alto, CA, USA) version 15.6 using the Acuros XB algorithm for final dose calculation. Acuros XB is also a type C algorithm. The commissioning of Varian accelerators in the TPS was described in Refs. 5, 10 An in‐slice grid size of 2.5 mm was used for the calculations except for spine mets and brain SRT, where the grid size was 1.5 mm. Dose was reported as dose‐to‐medium. For each case class, one of five different standard treatment techniques (static‐conformal, IMRT, FFF‐IMRT, FFF‐SBRT, and VMAT) was used for treatment planning. For all IMRT and VMAT plans, portal dose measurements using the on‐board imager were performed as part of the patient‐specific QA. The acceptance criterion was 95% of points fulfilling gamma < 1, with a dose difference (DD) < 3% and distance‐to‐agreement (DTA) < 2 mm. All plans fulfilled this criterion.

### 2.2 Mobius3D

Dose calculation in M3D is based on a convolution/superposition algorithm using beam models that are pre‐commissioned with standard reference datasets for the different beam energies of Varian TrueBeam machines. M3D offers options to customize its stock reference beam models to the user's individual treatment machines.^11^ We followed the vendor's recommended procedure and confined the adaptation of the beam models to dose–monitor‐unit calibration and Dosimetric Leaf Gap (DLG) correction.^4^, ^12^ An isotropic voxel grid with a grid size of 3 mm was used for M3D calculations, except for H&N with an isotropic voxel size of 2.5 mm, and spine mets and brain SRT, with an isotropic voxel size of 2.0 mm. The voxel sizes were chosen automatically by M3D. The dose‐to‐material calibration cannot be set.

### 2.3 SciMoCa

Dose calculation in SMC is based on a Monte Carlo algorithm.^3^, ^5^, ^6^ The vendor offers pre‐commissioned beam models that were derived from pooled data (generic beam models) and fully commissioned, customized beam models that are adapted to individual treatment machines by the vendor.^3^, ^5^, ^6^ Customized beam models for the three beam modalities 6 MV, 6 MV FFF, and 15 MV were commissioned on the basis of 14 depth dose curves (10 × 10 mm^2^–400 × 400 mm^2^), output factors, and cross‐profiles of the maximum field size. These measurements were largely identical to the base data of the TPS, apart from some small field measurements that were not required for TPS commissioning. The physical leaf positions were calibrated for both beam model types to the same value from a set of standard DLG measurements used to commission the TPS. An isotropic voxel size of 2.5 mm was used except for spine mets and SRT cases, where the voxel size was 1.5 mm. The statistical uncertainty was chosen such that the volume receiving at least 70% of the maximum dose had an uncertainty < 0.5% for the total treatment plan dose. Dose was reported as dose‐to‐medium.

### 2.4 Dose comparison

The 3D dose distributions from M3D and SMC were compared to the respective Acuros XB dose distributions using a global gamma evaluation^13^ with exclusion of doses below 5% of the maximum dose. The percentage of points fulfilling gamma < 1, that is, the gamma pass rate (GPR), was denoted as GPR (DD, DTA), where a number of different DD and DTA settings were used: GPR (5%, 3 mm), GPR (3%, 3 mm), GPR (2%, 2 mm), and GPR (2%, 1 mm). Furthermore, the difference in mean target dose (ΔD) versus Acuros XB was calculated. Dose–volume metrics were not investigated, since the 20 case classes varied so much in their treatment goals that a meaningful common parameter was not available.

Comparison of M3D versus Acuros XB was performed in the Mobius3D software, while the SciMoCaCompare module of the SciMoCa software (Radialogica LLC, St. Louis, MO, USA) was used for comparisons of SMC. Since M3D does not support dose export, it was not possible to compute GPR of M3D and SMC by the same algorithm.

### 2.5 Determination of action level

For the criteria ΔD and the four GPRs, action levels were determined that resulted in approximately *q* = 0.02, 0.05, 0.1, and 0.2 plan failure rate for each QA option by computing the percentiles and rounding to the next multiple of 0.5 and 5, respectively. Since GPR can be insensitive to target dose deviation, the criteria ΔD and GPR at *q* = 0.1 and 0.2 failure rate were also combined, that is, a plan fails if it fails according to ΔD AND GPR. Failing plans for either criterion were investigated for common causes.

The report of AAPM TG 218^14^ gives an alternative recommendation for establishing the action interval 2A from process observations (Equation 3):

$$2A=\beta \sqrt{\sigma^{2}+{\left(\bar{x}-T\right)}^{2},}$$

where T is the QA target value (e.g., GPR = 100), and $\sigma^{2}$ and $\bar{x}$ are the observed variance and mean of the QA results. For the sake of clarity, the action levels would be T−A and T+A for a two‐sided error and T−A for a one‐sided error like GPR. We can think of the root as the nominal variability of the QA process in the presence of systematic and random errors. In case the systematic component (from both ISDC and TPS) becomes very small, the root converges to the standard deviation of the QA process. Then, A becomes a β/2‐fold multiple of this standard deviation. Assuming a Gaussian distribution of x, q, and β/2 can be related via the normal distribution function, see Table 1. Notice that since the distribution of GPR values is truncated at 100, these values differ from those of a two‐sided distribution like that of ΔD. Notice further that in the limit of vanishing systematic errors, the (tentative) TG 218 recommendation of *β* = 6 amounts to the stipulation that a QA failure is a 3‐sigma event. To investigate the assumption of normal error distribution within the TG 218 formalism, we compared the nominal and observed failure rates for various action levels.

**TABLE 1:** Relationship between QA failure rates *q* and factors *β*/2 according to the TG 218 formalism for the action level and assuming a normally distributed error of the QA process.

*q*	0.0014	0.0027	0.02	0.023	0.046	0.05	0.1	0.2
*β*/2 for GPR	3	−	2.05	2	–	1.65	1.28	0.84
*β*/2 for ΔD	−	3	2.33	–	2	1.96	1.65	1.28

*Note*: Since the distribution of GPR values is right truncated, while the distribution of difference of the mean dose ΔD is two‐sided, the values of β/2 differ.

## 3 Results

**FIGURE 1:**
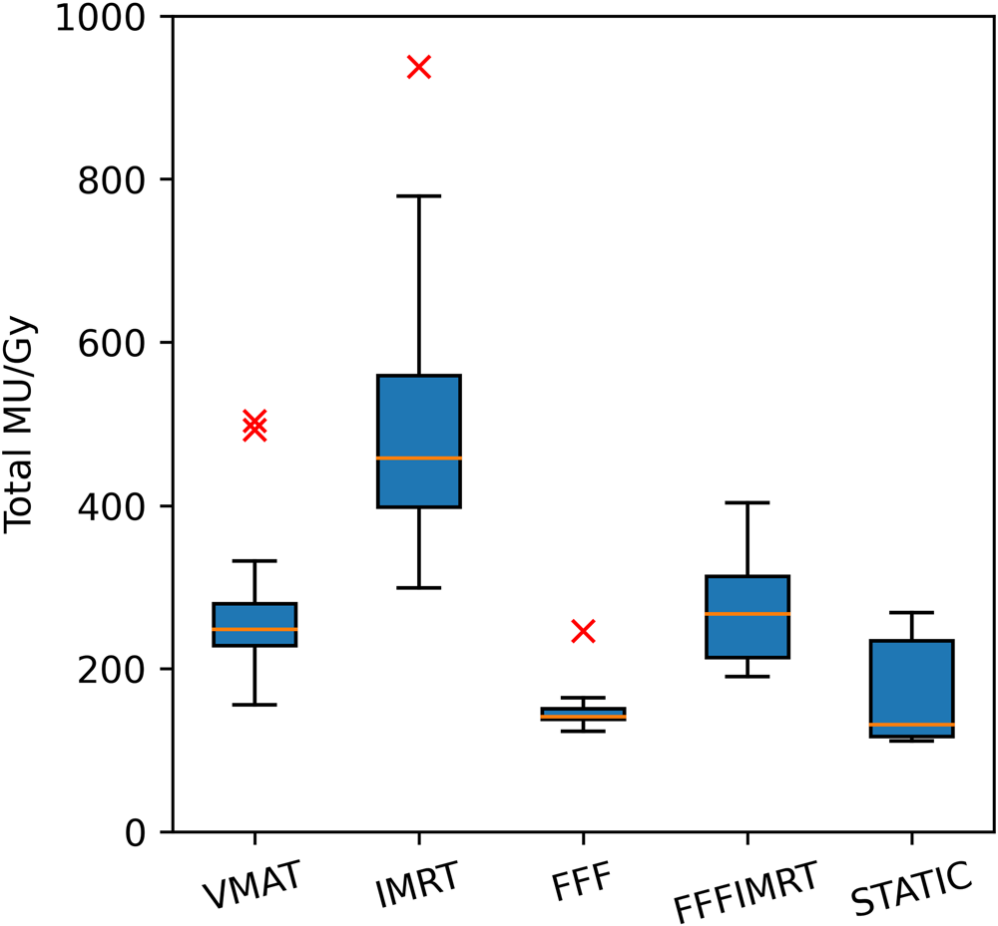
Modified box‐and‐whisker plot of the monitor unit expenditure (in terms of total MU/Gy) by treatment technique. Red line: median. Extents of box: central 50% of data points = interquartile range. Whiskers: min/max values within 1.5‐fold interquartile range. Red crosses: outliers.

Figure 1 shows an overview of the monitor unit expenditure for the five treatment techniques. Of the 20 case classes, 2 were treated with 6FFF stereotactic conformal beams (lung‐SBRT/static‐conformal and brain SRT), 1 was treated with 6FFF IMRT (lung‐SBRT/IMRT), 4 were treated with static conformal fields (breast, breast‐supraclav+DIBH, breast‐SIB, and palliative), 4 were treated with static beam IMRT (lung, arm, leg, and esophagus), and the remaining 9 with VMAT.

**FIGURE 2:**
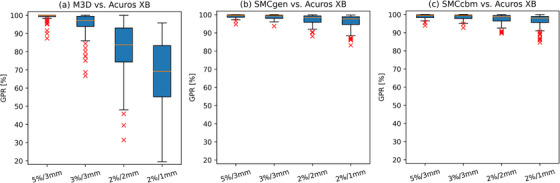
Modified box‐and‐whisker plots of the gamma pass rates GPR (DD, DTA) for different combinations of dose difference (DD) and distance‐to‐agreement (DTA) for (a) M3D, (b) SMCgen, and (c) SMCcbm versus Acuros XB, respectively. Red line: median. Extents of box: central 50% of data points = interquartile range. Whiskers: min/max values within 1.5‐fold interquartile range. Red crosses: outliers.

The GPR results for the four DD/DTA criteria combinations are shown in Figure 2.

**FIGURE 3:**
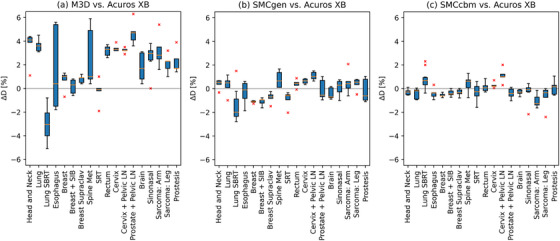
Modified box‐and‐whisker plots of the difference of mean target dose ΔD for (a) M3D, (b) SMCgen, and (c) SMCcbm versus Acuros XB, differentiated per case class. Positive values indicate the ISDC dose is higher than the TPS dose. Red line: median. Extents of box: central 50% of data points = interquartile range. Whiskers: min/max values within 1.5‐fold interquartile range. Red crosses: outliers.

The results of the difference of mean target dose ΔD are displayed in Figure 3. The mean ΔD ± 1 SD across all case classes was 1.75% ± 2.41% for M3D, −0.17% ± 1.03% for SMCgen, and −0.11% ± 0.78% for SMCcbm.

**FIGURE 4:**
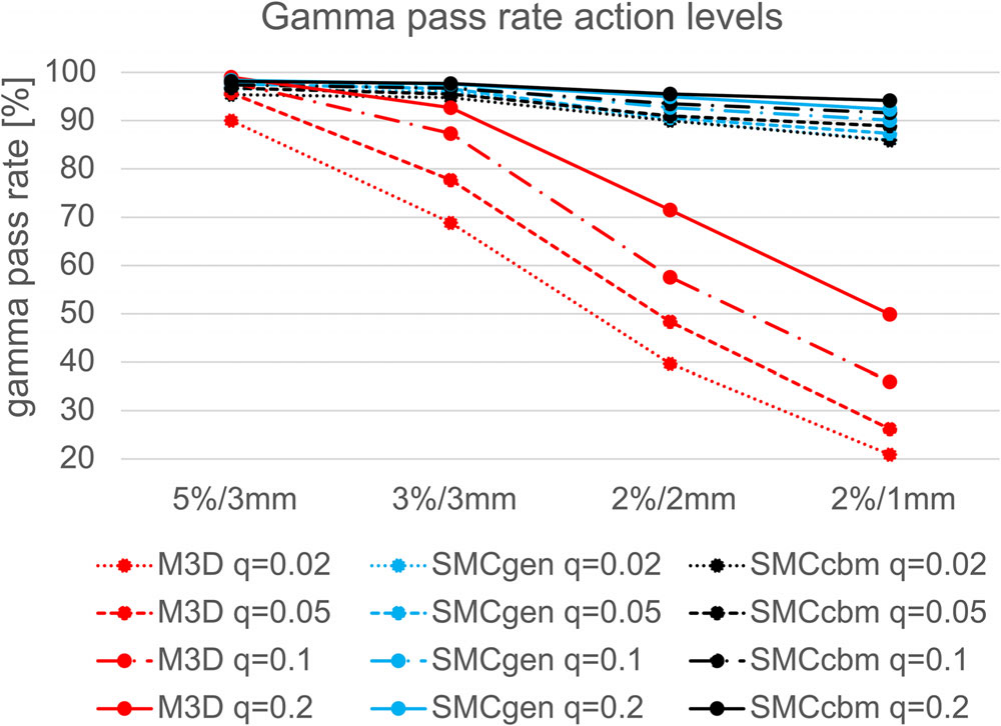
Action level in terms of gamma pass rate for each of the four gamma criteria as a function of test failure rate *q* for M3D (red), SMCgen (blue), and (c) SMCcbm (black) versus Acuros XB. Failure rates were *q* = 0.02 (dotted), *q* = 0.05 (dashed), *q* = 0.1 (dot‐dashed), and *q* = 0.2 (solid).

**FIGURE 5:**
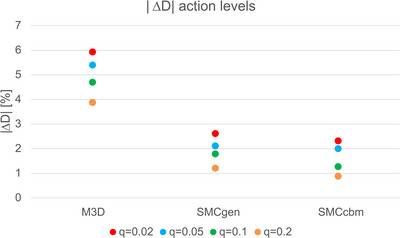
Action level in terms of the absolute difference in mean target dose |ΔD| depending on test failure rate *q* for M3D (left), SMCgen (center), and (c) SMCcbm (right) versus Acuros XB. Failure rates were *q* = 0.02 (red), *q* = 0.05 (blue), *q* = 0.1 (green), and *q* = 0.2 (orange).

From these findings, the action levels as a function of QA failure rate *q* were determined. For the four different gamma criteria, the results are shown in Figure 4. The action levels for the absolute difference in mean target dose |ΔD| are shown in Figure 5.

For the analysis of the effectivity of the combined application of both evaluation criteria, GPR and absolute difference in mean target dose |ΔD|, action levels 1 and 2 were determined for *q* = 0.1 according to the conversion factors of Table 1 and rounded to the next multiple of 5 and 0.5, respectively (see Table 2). Action level 3 is a further restriction over action level 2, being determined for *q* = 0.2, and is added for better analysis of the difference between generic and custom beam models.

**TABLE 2:** Action levels AL1, AL2 for *q* = 0.1 failure rate and AL3 for *q* = 0.2 failure rate for the combined criteria analysis.

Action level (derived from ISDC)	1 (M3D)	2 (SMCcbm)	3 (SMCcbm)
|ΔD|	≤ 4.5%	≤ 1.5%	≤ 1.0%
Gamma	GPR (3,3) ≥ 90%	GPR (2,2) ≥ 95%	GPR (2,1) ≥ 95%

**FIGURE 6:**
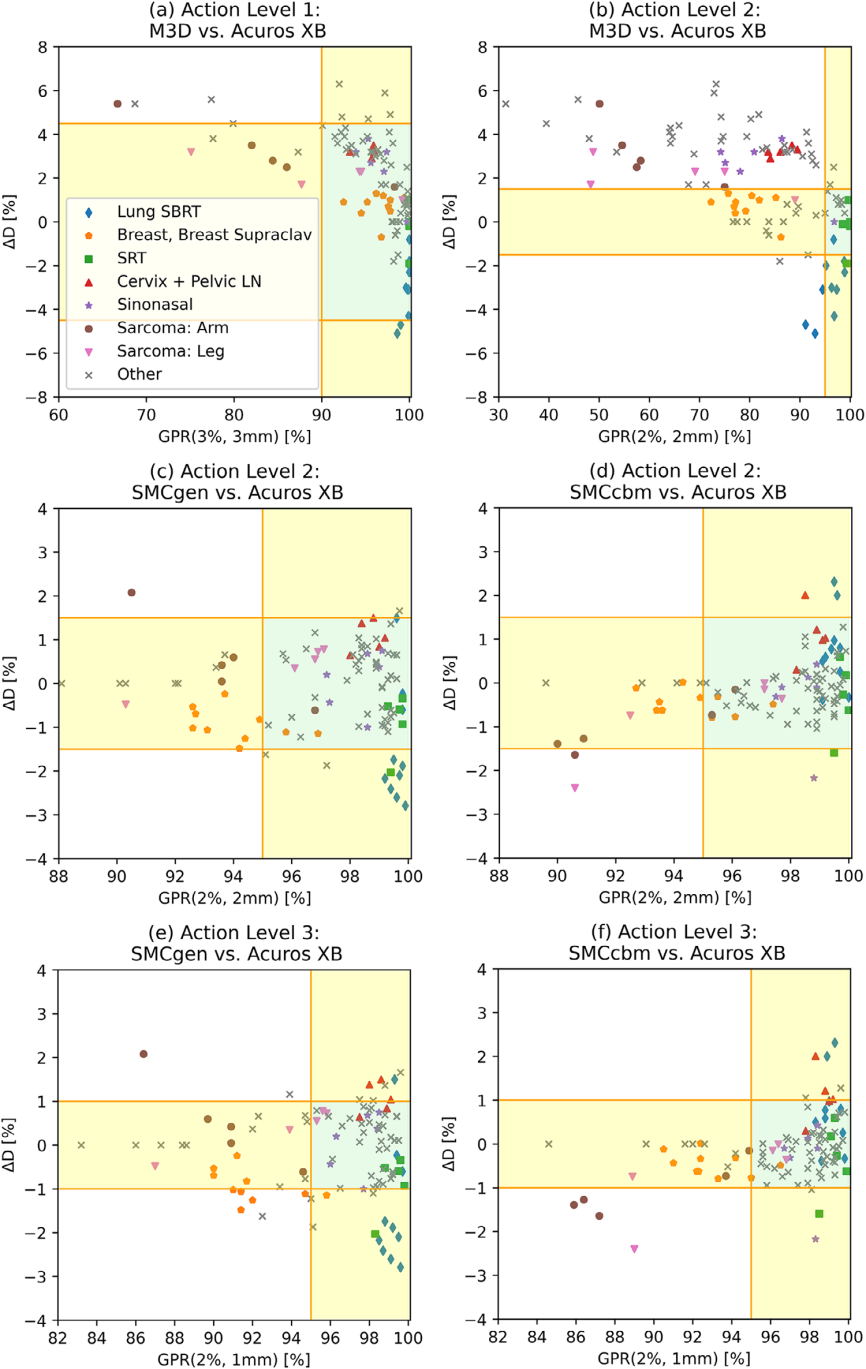
Scatter plots of combined criteria scores of each case for M3D with action level 1 (a), M3D with action level 2 (b), SMCgen with action level 2 (c) and action level 3 (e), and SMCcbm with action level 2 (d) and action level 3 (f). The green zone denotes the area where both criteria, gamma pass rate and absolute difference in mean target dose |ΔD|, are fulfilled. The yellow zones denote the areas where only one criterion is fulfilled. White zones denote the areas where none is fulfilled.

The distributions for the combined criteria with respect to these action levels are shown in Figure 6 and detailed in Table 3. In the same table, we show the failure rates that would be expected from the TG 218 formalism for these action levels, assuming a Gaussian distribution of QA results as in Table 1.

**TABLE 3:** Pass/Fail rates for the combined application of both criteria, gamma pass rate and absolute difference in mean target dose |ΔD|, for M3D with action level 1 and action level 2, and SMCgen and SMCcbm with action level 2 and action level 3.

ISDC action level	M3D 1	M3D 2	SMCgen 2	SMCcbm 2	SMCgen 3	SMCcbm 3
**Passed both**	82%	13%	70%	80%	53%	69%
**Failed both**	3%	56%	1%	2%	8%	4%
**Failed gamma**	11%	78%	19%	15%	29%	22%
**Failed |ΔD|**	10%	65%	12%	7%	26%	13%
**Expected fail rate from AL gamma**	11 ± 3.3%	42 ± 6.5%	10 ± 3.2%	8 ± 2.8%	18 ± 4.2%	14 ± 3.7%
**Expected fail rate from AL |ΔD|**	13 ± 3.6%	62 ± 7.9%	13 ± 3.6%	5 ± 2.2%	31 ± 5.6%	17 ± 4.1%

*Note*: The two bottom rows give the predicted failure rate via the TG 218 formalism, assuming a Gaussian distribution of QA results. Errors of expected failure rates are binomial estimates.

## 4 DISCUSSION

This investigation aimed to quantify the benefit of accuracy for different ISDC concepts in terms of sensitivity at a constant workload, that is, a failure rate. Since the measurement of sensitivity requires a set of test cases with known or stipulated QA status, it always contains a degree of arbitrariness. We chose our approach because it affords greater independence from the set of test cases and relies only on the assumption that tighter QA criteria would in any case increase sensitivity to less than obvious errors—at a controlled failure rate. In addition, our method does not rely on assumptions about the distribution of QA results but determines action levels solely from percentiles of the results for a large number of cases, that is, by counting. This makes the method robust but perhaps too cumbersome for general usage.

An alternative approach to arrive at action levels was presented in the report of AAPM TG 218.^14^ It requires a smaller sample size but invokes assumptions about the distribution of QA results. The action levels depend on the choice of a parameter β, which is somewhat elusive. However, under certain assumptions, β can be related to the failure rate *q* via a Gaussian distribution function. We tested this hypothesis because the TG 218 approach could provide a shorter route to institution‐specific action levels than ours, potentially requiring fewer cases.

We found that for QA criteria that are two‐sided (ΔD), the failure rate, as expected from the action level, matches the observed failure rate in 6 out of 6 cases within uncertainties (Table 3). The agreement was much worse (1 out of 6) for the one‐sided GPR criterion, particularly when the mean GPR was close to 100%. In this case, the error distribution becomes very asymmetrical with a heavy tail and is therefore not well approximated by a Gaussian. For a distribution of GPR like those for SMC with mean GPR ± standard deviation between 96% ± 2% and 98% ± 1.4% it seems to be possible to correct β/2 by a factor 1.45, which is likely influenced by the mix of test cases. A more thorough investigation is beyond the scope of this manuscript.

Irrespective of the approach, both methods establish a link between action level and failure rate for a particular combination of ISDC and TPS (each with its own particular quality of commissioning). Hence, the pair of action level/failure rate can serve as an inter‐institutional measure of the overall quality of treatment planning and its QA.

In our investigation, the gamma criterion applied to the entire extent of the dose distribution and therefore lacks specificity, especially for small target volumes. This is the reason why we also considered a target‐centric parameter, the difference between the mean target dose of the ISDC and the TPS in parallel with gamma. Also, the GPR is affected by Monte Carlo statistical noise,^15^ while the mean dose is the most robust parameter against such noise. It has been shown^3^ that the effect of a 0.5% statistical uncertainty on GPR with a small DTA is rather small, in the order of < 1.5% variation of GPR, which is the same magnitude as the variability of different implementations of the method.^15^ Naturally, more case class specific criteria like region‐of‐interest restricted gamma analysis or dose‐volume parameters would be used in practice but were not at our disposal here as they would not allow us to view many different case classes simultaneously.

The central results of our study are given in Table 3 and Figure 6. At its ideal action level of GPR (3,3) ≥ 90%, |ΔD| ≤ 4.5%, M3D fails in both criteria in 3% of the cases. These cases were highly modulated IMRT treatments (two esophagus and one arm sarcoma). In terms of |ΔD| patterns of failure, there is a clear finding that lung SBRT cases, treated with 6FFF‐IMRT beams, consistently scored a lower dose by M3D; a finding which has been reported previously.^16^ With respect to gamma failures, there seems to be a tendency towards lower GPR for arm sarcoma cases treated again with 6MV‐IMRT. With the more stringent action level 2, the failure rates rise substantially, with 56% failing both criteria. There seems to be no apparent patterns or clusters of cases for outliers.

Several studies that evaluated the agreement of M3D with TPS and measurements also resulted in GPR (3,3) around 90%–95%,^17^, ^18^, ^19^ which is in keeping with our results. The bias towards positive ΔD in Figure 3a suggests that the DLG value is too high. However, the static beam cases also present with a higher average mean DD, the spread between the highest and lowest ΔD is rather symmetrical, and the deviation is driven primarily by VMAT cases, even though IMRT cases have a higher monitor unit expenditure (Figure 1). Difficulties with finding a general DLG value, that is, optimum for both VMAT and IMRT have been reported.^20^ All of this suggests that the deviation of the mean ΔD from 0 is multi‐factorial.^21^

At the action level 2 of GPR (2,2) ≥ 95% and |ΔD| ≤ 1.5%, the Monte Carlo ISDC fails in both criteria in 1% (generic beam model) and 2% (custom beam model) of cases. In the single‐criteria and both‐criteria pass rates, the custom beam models score somewhat better than the generic models (Table 3 and Figure 3b,c).

**FIGURE 7:**
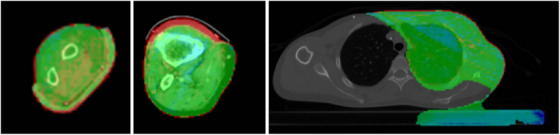
Volumes of low gamma pass rate (GPR) for three cases computed with SMC. The arm (left) and leg (center) sarcoma cases show a higher SMC dose at the patient outline (red area in the gamma plot). This stems from a different handling of the patient outline in the density models of Eclipse and SMC. In the middle image, only SMC computes dose in a dummy volume that extends outside the patient outline. The patient fixation of the breast case (right) shows a lower dose by SMC, which originates from a different translation of Hounsfield values into material properties by Eclipse and SMC (dose difference occurs in the foam core).^5^

Both SMC computations have in common that breast and arm sarcoma cases present with lower GPR. This can, in fact, be traced back to a difference in the patient density model between Acuros XB and SMC. Figure 7 gives three examples that show the location of the low GPR volumes. On the left is an arm sarcoma case that illustrates a difference in the handling of the patient outline, which contains more density‐filled voxels in SMC than in Eclipse. This is due to a different interpretation of partially filled voxels at the patient outline. The middle image is a leg sarcoma case with a dummy contour extending into the space between the fixation device and the leg. The difference in dose computation is limited to the dummy contour, in which SMC computes dose in air while Eclipse does not. Finally, the right image shows a breast case where the immobilization device and patient support structures are included in the density model but have a different material composition in SMC versus Eclipse.^5^ To a large extent, the drop in GPR could be avoided if the gamma distribution was not evaluated over the entire dose calculation volume. This also underlines the complementary nature of the comprehensive, spatially resolved, yet unspecific gamma analysis and the compounded, yet target‐focused mean target dose.

A further case class with consistent discrepancies could be found for SMCgen, where all five lung SBRT‐IMRT cases presented with a lower mean target dose than in Eclipse; this could not be observed for SMCcbm, that is, is caused by the beam model. In fact, these five cases were the only application of 6FFF‐IMRT, so that this observation could be attributed to the generic beam model, which did not seem to be a good fit for the actual beam properties. In case such systematic discrepancies are observed, the issue may only be resolved by well‐designed measurements specific to the hypothetical cause.

To investigate the performance of the generic beam models further, the SMC options were evaluated with the stricter action level 3, see Table 3. Here, the performance gap between the custom and the generic models widened compared to action level 2. The percentage of cases passing both criteria were 69% and 53% for custom and generic models, respectively. Even if the problematic 6FFF model had been removed from the analysis, a gap of 11% remained. For SMCcbm, only 4% of cases failed both criteria, while 8% failed both criteria for SMCgen. The two ISDC had only one failed case (an arm sarcoma) in common, whose cause for failure could be traced back to the aforementioned differences in the patient model. Even though the overall performance of generic beam models was not markedly different from custom beam models for action level 2, the benefits of custom beam models begin to show at action level 3, where SMCgen presents twice as many QA fails than SMCcbm, or in other words: gratuitously doubles the workload. We conclude that it cannot be taken for granted that a generic model is always a sufficiently good match for a specific beam, as in case of the 6FFF model here. This obvious assertion also applies to the generic models of M3D, where there was no evidence that any one of the three models performed better than the others.

The observable difference between individually commissioned and generic beam models also gives an indication of the magnitude of commissioning errors of the TPS that may be picked up by SMC at action level 3. The criterion GPR (2,1) of action level 3 was also established as optimal in conjunction with a Monte Carlo TPS via a receiver‐operator analysis,^3^ using custom beam models for Elekta accelerators. We interpret this as evidence that the typical accuracy of TPS with different flavors of a type C dose algorithm is indeed similar. It was also found that the criterion GPR (2,1) can be satisfied by SMCcbm in comparison to measurements with detector arrays.^6^, ^22^

The majority of published validations of ISDC arrived at the conclusion that a GPR (3,3) > 90%–95% or GPR (3,2) > 90%–95% represents a satisfactory agreement to the TPS.^17^, ^18^, ^19^ The report of AAPM TG219^2^ suggested the criterion of GPR (3,2) > 95%. These recommendations stem from a patient‐centric, clinical view, that is, would avoid *random* serious mistreatments. The recent report of AAPM TG360^23^ pointed out that TPS failure modes such as incorrect output factors or MLC modeling are dominating sources of errors of PSQA, which ties in with previous audit findings.^24^ This suggests that *systematic* TPS inaccuracies play a significant yet overlooked role in PSQA. These systematic inaccuracies often originate from errors in the base data for commissioning the TPS and the ISDC. A recent paper by Kowatsch et al.^25^ investigates the causes, severity and frequency of various error types present in the base data submitted for SMC custom beam modeling, emphasizing the benefit of independent commissioning. From our study, it appears that a GPR (2,1) > 95% is a suitable criterion for scanning for commissioning errors in either ISDC or TPS and should not result in failure rates much > 20%. While this failure rate may be acceptable for screening for systematic errors, it is clearly not acceptable for PSQA. In combination with other criteria, it may become more workable if we assume that a well‐commissioned type C TPS (like the Acuros XB here or Monaco^3^) and a well‐commissioned type C ISDC like SMCcbm operate at the same level of accuracy, action level 3 produces a background of 4% PSQA failures, of which 2% may be due to the TPS and 2% may be due to the ISDC. In contrast, at action level 2, M3D produces 27 times more PSQA failures than SMCcbm (Table 3).

It appears that the findings of TG360 call for an ISDC whose accuracy matches or exceeds that of the TPS to be surveyed. This is just another way of restating the recommendation of the ICRU83 report.^1^ The report of TG360^23^ also lends a different angle to the utilization of generic beam models for ISDC, which obviously require a thorough assessment of whether they match the specific beam properties, especially output factors and MLC modeling, for example, correct leaf position calibration.

Summarizing, we find that higher accuracy of an ISDC enables stricter acceptance criteria without increasing the workload. To capture also systematic errors of TPS commissioning in addition to random gross treatment plan errors, the action level needs to be adjusted to the capabilities of the TPS. For type C dose calculation TPS, a PSQA action level of GPR (2,1) > 95% OR |ΔD| < 1% or stricter is feasible. Generic beam models require strict verification of their agreement with individual beam properties to guarantee this level of performance. Failure to achieve high pass rates in excess of 95% at the above action level may indicate that the beam models of ISDC or TPS need attention.

## 5 CONCLUSION

Monte Carlo dose calculation provides a significant benefit for ISDC as patient‐specific quality assurance, allowing substantially more stringent acceptance criteria than an analytical algorithm. The performance of generic beam models can match that of customized beam models, but they still need to be validated against the individual beam properties. Apart from dose calculation, the generation of the patient density model is a source of substantial variability. This is one cause of low gamma pass rates, which could be ameliorated by restricting gamma analysis to the most relevant volumes. Depending on the quality of the commissioning of the TPS, a gamma (2%, 1 mm) pass rate > 95% is a feasible choice for Monte Carlo based ISDC with custom beam models.

## AUTHOR CONTRIBUTIONS

Lone Hoffmann and Ditte Sloth Møller conceived the study and wrote the manuscript. Mai‐Britt Linaa conducted data collection.

## CONFLICT OF INTEREST STATEMENT

The authors declare no conflicts of interest.

## Footnotes

1 Type B algorithms are typically analytical and consider density variations by corrections to pre‐computed electron kernels in water. Type C algorithms employ explicit electron transport in‐situ and are typically either Monte Carlo methods or Linear Boltzmann equation solvers.
